# Effects of Fulvic Acid on Growth and Nitrogen Utilization Efficiency in M9T337 Seedlings

**DOI:** 10.3390/plants12233937

**Published:** 2023-11-22

**Authors:** Bo Yu, Laiping Wang, Dongdong Cui, Wensheng Gao, Xiaomin Xue, Peixian Nie

**Affiliations:** 1 Shandong Institute of Pomology, Tai’an 271000, China; 2018200076@stu.syau.edu.cn (B.Y.); wanglaiping666@163.com (L.W.); cuidd198212@163.com (D.C.); xuexiaomin79@126.com (X.X.); 2 College of Horticulture, Shenyang Agricultural University, Shenyang 110866, China; 3 Shandong Provincial Department of Agriculture and Rural Affairs, Shandong Agricultural Technology Extension Center, Jinan 250100, China; gaowensheng@sina.com

**Keywords:** apple, fulvic acid, photosynthesis, C and N metabolism, NUE

## Abstract

Both fulvic acid (FA) and nitrogen (N) play important roles in agricultural production in China. Plants typically show a higher nitrogen utilization efficiency (NUE) under FA application. However, the role of FA application in apple growth and NUE remains unclear. A hydroponic culture experiment was performed, and M9T337 seedlings (a dwarf apple rootstock) were used as the experimental subjects. The biomass, photosynthesis, accumulation, and distribution of photosynthates, N absorption and assimilation, and relative gene expression in the seedlings were examined after treatment with five different concentrations of FA (0, 60, 120, 180, and 240 mg·L^−1^, represented by CK, FA1, FA2, FA3, FA4, respectively). The results showed that the seedling dry weight and ^15^NUE were enhanced by FA, and both were highest under the FA2 (the concentration of fulvic acid is 120 mg·L^−1^) treatment. Further analysis revealed that under the FA2 treatment, the root morphology was optimized, and the root activity was relatively high. Compared with CK (control, the concentration of fulvic acid is 0 mg·L^−1^), the FA2 treatment strengthened photosynthesis, elevated the key enzyme activities related to C metabolism, upregulated the gene expression of sugar transport proteins, and increased the root sorbitol and sucrose contents, which suggested that the FA2 treatment optimally affected the root growth and N absorption because it enhanced photosynthate synthesis and the leaf-to-root translocation of photoassimilates. The seedlings in the FA2 treatment group also showed a significantly higher NO_3_^−^ influx rate and *NRT* (nitrate transporter) gene expression in the roots. Moreover, relatively high N metabolism-related enzyme activities in the leaves and roots were also observed under the FA2 treatment. The isotope labeling results showed that the optimal FA2 supply not only promoted seedling ^15^N absorption but also optimized the distribution of C and N in the seedlings. These results suggested that an optimal FA supply (120 mg·L^−1^) enhanced seedling NUE by strengthening photoassimilate synthesis and transport from leaves to roots, regulating N absorption, assimilation, and distribution.

## 1. Introduction

N, an important and widely used nutrient element in modern agriculture, strongly contributes to the promotion of fruit quality and yield and has become a central component of apple orchard nutrient management [1,2]. Recently, excessive N fertilizer input has become a common phenomenon in apple orchards in China, and the N applied to orchards is not utilized efficiently, which may not only decrease the motivation of fruit farmers due to increased orchard production costs but also result in a series of ecological and environmental problems, hindering the green and high-quality development of the apple industry [3]. Improving the efficiency of N utilization has become a very urgent problem to be solved in the main apple orchard production areas. Therefore, the new fertilization methods should be studied with great efforts.

To date, substantial research advances have been made to explore effective solutions for enhancing the N utilization efficiency (NUE) of apple orchards in China [4,5,6,7]. As a reactive humic acid substance, fulvic acid (FA) plays an important role in modern agricultural production [8,9]. For example, Liu et al. [10] observed that FA application obviously elevated the yield of winter wheat. Chen et al. [11] reported that the negative effects of cadmium (Cd) stress on lettuce could be significantly mitigated by the elevation in the Cd stress tolerance induced by the application of exogenous FA under Cd stress. N is indispensable for the development of modern agriculture in China, and many studies have been performed to explore the positive effects induced by FA application on plant NUE. Similar to the results obtained by Kong et al. [12] in which humic acid (HA) application reduced N loss, authors such as Gao et al. [9] also reported that FA application could efficiently reduce ammonia volatilization from urea by inhibiting urease activity, and the formation of stable FA urea complexes under the combined application of FA and urea could also delay urea decomposition. Due to regulations, the goal of N preservation and N loss reduction has been achieved, which not only minimizes adverse environmental effects but is also beneficial in improving plant NUE. Apart from the positive effects observed in soil characteristics, the changes in plants induced by FA application are also beneficial for the promotion of plant NUE. Authors such as Canellas et al. [13] reported that FA application could simulate root elongation and optimize root morphology, which can enhance the N absorption ability of plants. Moreover, FA application could also improve plant NUE by enhancing carbon/nitrogen metabolic processes [9,14]. Therefore, FA is widely used as an N fertilizer synergist in agricultural production. Owing to its unique economic value as well as its important roles in revitalizing the rural economy, the apple has become one of the most important extensively cultivated fruit trees in China [2]. Enhancing apple tree NUE is critical to the high-quality development of the apple industry. In contrast, specifics regarding the effects of FA application on apple plant NUE remain unclear, particularly from the perspective of C and N metabolism regulation.

There are strong interactions between C and N metabolism, which exist in metabolic processes and energy levels, and the synchronized coordination of C and N metabolism is of great importance for improving plant growth and nutrient absorption [15,16,17]. Ren et al. [18] reported that the growth inhibition of maize induced by environmental (drought) stress could be obviously reduced through the maintenance of plant C and N metabolism induced by exogenous melatonin under stressful conditions. Xu et al. [4] also found that the elevation in apple seedling NUE was mostly attributed to the promotion of photosynthetic C assimilation efficiency and N metabolism-related enzyme activities under the application of an appropriate and constant potassium (K) supply. Therefore, exploring the changes in the C and N metabolism of apple plants under FA-treated conditions may help clarify the effects of FA on apple plant growth and NUE. This study was performed in a growth chamber using hydroponics. M9T337 seedlings were selected as the experimental material, and five treatments (different FA supply levels) were employed to evaluate the effects of FA application on photosynthesis, C metabolism, nitrate (NO_3_^−^-N, the only N source in this experiment) absorption, and utilization of M9T337 seedlings. The results of this trial will help us comprehend the physiological mechanism of FA that regulates apple NUE and provide a new method for the improvement in the NUE in apple orchards.

## 2. Results

### 2.1. Plant Biomass and Root Morphology of Seedlings

**Figure 1:**
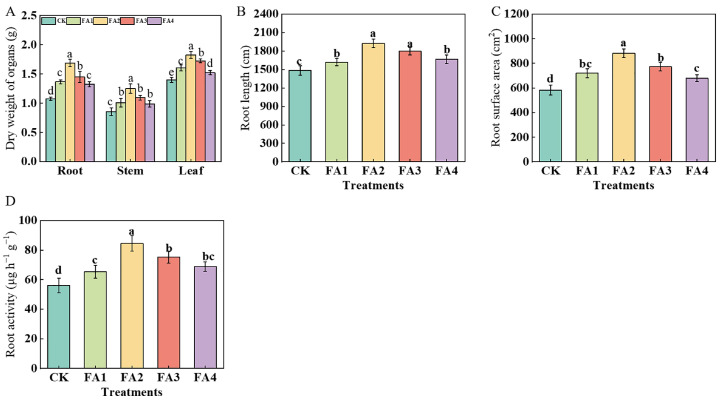
Organ dry matter weight (**A**), total root length (**B**), surface area of seedlings (**C**), and root activities (**D**) treated with CK (0 mg·L^−1^ fulvic acid), FA1 (60 mg·L^−1^ fulvic acid), FA2 (120 mg·L^−1^ fulvic acid), FA3 (180 mg·L^−1^ fulvic acid), or FA4 (240 mg·L^−1^ fulvic acid). Vertical bars on the histograms indicate standard deviations of three replications. Different letters indicate statistically significant differences (*p* < 0.05).

As shown in Figure 1A, the seedling leaf, stem, and root dry weights were obviously elevated under the FA treatment, and both were maximized under the FA2 treatment, i.e., 30.71% (leaf), 45.35% (stem), and 56.48% (root) higher than that in the CK, respectively. In addition, no significant differences were observed in the stem dry weight among the FA1, FA3, and FA4 treatments, and a similar tendency was shown in the root dry weight between the FA1 and FA4.

After FA application for 30 days, the total root length of the seedlings under various FA levels was higher than that of the CK (Figure 1B). Similar to the changes in the total root length, the root surface area of the seedlings also showed a significant elevation under the FA treatment, and the highest root surface area was observed under the FA2 treatment, which was increased by 51.46% compared with the CK (Figure 1C). Moreover, the root activities also differed significantly under various treatments. The highest value was observed under the FA2 treatment, and the lowest was found in the CK, which was only 0.66 times that of the FA2 treatment (Figure 1D).

### 2.2. Plant C Metabolism

#### 2.2.1. Photosynthetic Characteristics

**Figure 2:**
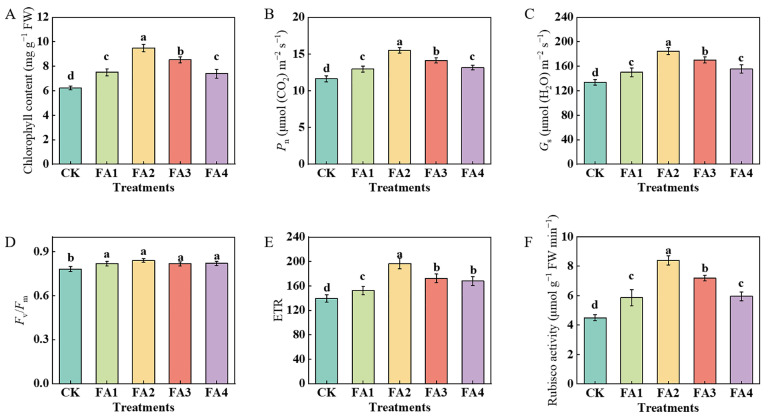
Chlorophyll content (**A**), *P*_n_ (**B**), *G*_s_ (**C**), *F*_v_/*F*_m_ (**D**), *ETR* (**E**), and Rubisco activity (**F**) in leaves treated with CK (0 mg·L^−1^ fulvic acid), FA1 (60 mg·L^−1^ fulvic acid), FA2 (120 mg·L^−1^ fulvic acid), FA3 (180 mg·L^−1^ fulvic acid), or FA4 (240 mg·L^−1^ fulvic acid). Vertical bars on the histograms indicate standard deviations of three replications. Different letters indicate statistically significant differences (*p* < 0.05).

The results in Figure 2 show that the FA application obviously influenced the photosynthetic performance of the leaves. After the FA application for 30 days, compared with the CK, the total chlorophyll content, *P*_n_ (net photosynthetic rate), and *G*_s_ (stomatal conductance) were both enhanced under all FA treatments, and both were maximized under the FA2 treatment, i.e., 52.49% (chlorophyll content), 33.28% (*P*_n_), and 37.81% (*G*_s_) higher than those in the CK, respectively (Figure 2A–C). The values of the *F*_v_/*F*_m_ (the maximum photochemical quantum yield of PSII) and *ETR* (electron transport rate) were also measured. As shown in Figure 2D, although all of the FA treatments significantly elevated the value of the *F*_v_/*F*_m_, no obvious difference was observed in the value of the *F*_v_/*F*_m_ among the FA treatments. Compared with the CK, exogenous FA supply treatments both significantly elevated the value of the *ETR*, which initially increased and then decreased. The highest value was observed under the FA2 treatment and was 40.81% higher than that in the CK (Figure 2E). Moreover, compared with the CK, exogenous FA supply treatments significantly elevated the value of the leaf Rubisco (ribulose-1,5-bisphosphate carboxylase-oxygenase) activity, which initially increased and then decreased. The best promotion of the Rubisco activity was observed under the FA2 treatment, i.e., 86.67% higher than that in the CK (Figure 2F).

#### 2.2.2. Changes in the Activities of Enzymes Related to C Metabolism

**Figure 3:**
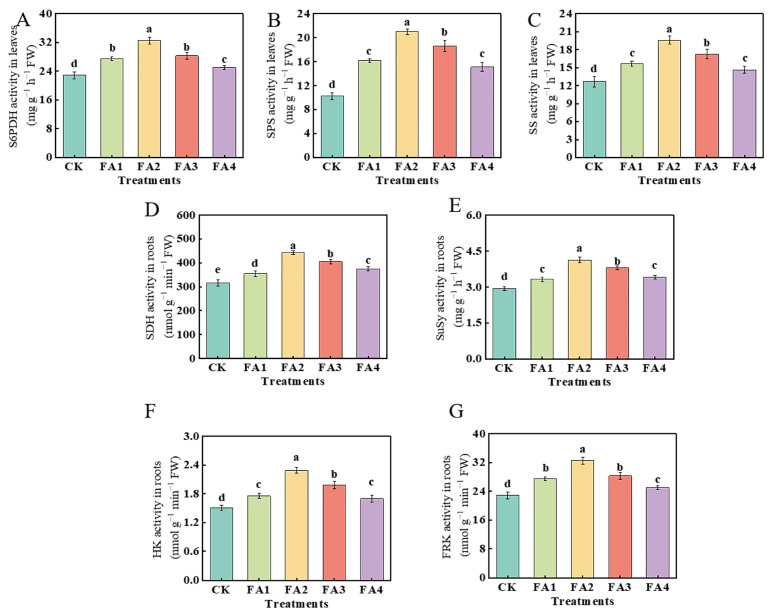
S6PDH, SPS, and SS activities in leaves (**A**–**C**) as well as the activities of SDH, SuSy, FRK, and HK in roots (**D**–**G**) treated with CK (0 mg·L^−1^ fulvic acid), FA1 (60 mg·L^−1^ fulvic acid), FA2 (120 mg·L^−1^ fulvic acid), FA3 (180 mg·L^−1^ fulvic acid), or FA4 (240 mg·L^−1^ fulvic acid). Vertical bars on the histograms indicate standard deviations of three replications. Different letters indicate statistically significant differences (*p* < 0.05).

As shown in Figure 3A, compared with the CK, the FA application (FA1–FA4) elevated the leaf S6PDH (sorbitol 6-phosphate dehydrogenase) activity to varying degrees. The highest elevation was observed under the FA2 treatment and was 42.41% higher than that in the CK. With the elevated FA supply concentration, the values of the SPS (sucrose phosphate synthase) and SS (sucrose synthase) activities both initially increased and then decreased, and both reached the highest level under the FA2 treatment (Figure 3B,C). In addition, the result of Figure 3D–F also showed that the activities of SDH (sorbitol dehydrogenase), SuSy (sucrose synthase), HK (hexokinase), and FRK (fructokinase) in the roots were also elevated under the FA treatments, and the FA2 treatment displayed the highest SDH, SuSy, and HK activities in the roots. The activity of FRK under both the FA1 and FA3 treatments was higher than that of the CK; however, there was no significant difference in the FRK activity between the FA1 and FA3 treatments.

#### 2.2.3. Sorbitol and Sucrose Contents

**Figure 4:**
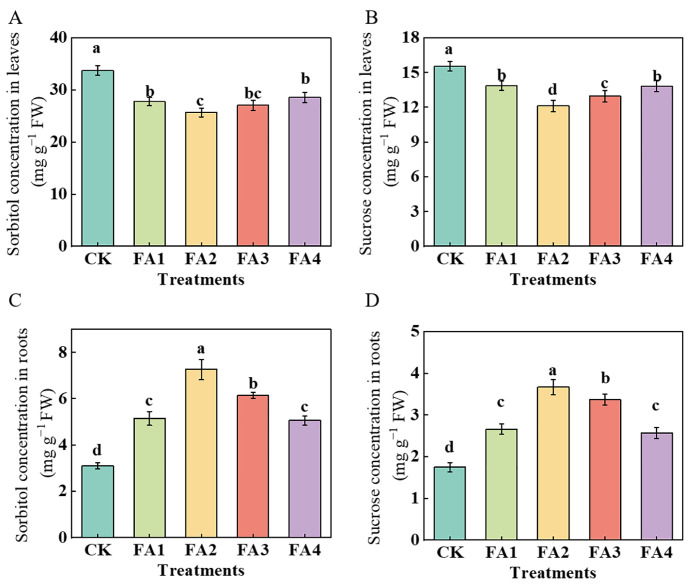
Sorbitol concentrations in seedling leaves (**A**) and roots (**C**) and sucrose concentrations in seedling leaves (**B**) and roots (**D**) treated with CK (0 mg·L^−1^ fulvic acid), FA1 (60 mg·L^−1^ fulvic acid), FA2 (120 mg·L^−1^ fulvic acid), FA3 (180 mg·L^−1^ fulvic acid), or FA4 (240 mg·L^−1^ fulvic acid). Vertical bars on the histograms indicate standard deviations of three replications. Different letters indicate statistically significant differences (*p* < 0.05).

In this study, we measured the sorbitol and sucrose contents in the leaves and roots, respectively. Regardless of the treatments employed in this study, the contents of both sorbitol and sucrose in the leaves were higher than those in the roots (Figure 4). As shown in Figure 4A,C, under the FA (FA1–FA4) treatment, the values of sorbitol were obviously decreased in the leaves and were elevated in the roots. The lowest values in the leaves and the highest in the roots both occurred under the FA2 treatment. For sucrose (Figure 4B,D), similar to the change in the sorbitol content, the contents of sucrose in the leaves under the various FA treatments were lower than those in the CK, and the lowest content was also observed in the FA2 treatment, i.e., 21.86% lower than that in the CK. Compared with the CK, the FA application (FA1–FA4) treatments elevated both the root sucrose contents and reached the highest level under the FA2 treatment, followed by the FA3 treatment.

#### 2.2.4. ^13^C Accumulation, Distribution, and Transport

**Figure 5:**
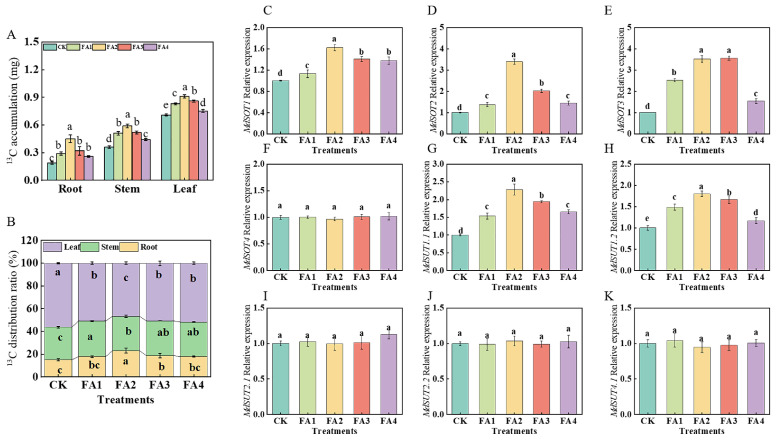
^13^C accumulation of seedlings (**A**), organ ^13^C distribution rate (**B**), and root *MdSOTs* and *MdSUTs* gene expression (**C**–**K**) treated with CK (0 mg·L^−1^ fulvic acid), FA1 (60 mg·L^−1^ fulvic acid), FA2 (120 mg·L^−1^ fulvic acid), FA3 (180 mg·L^−1^ fulvic acid), or FA4 (240 mg·L^−1^ fulvic acid). Vertical bars on the histograms indicate standard deviations of three replications. Different letters indicate statistically significant differences (*p* < 0.05).

After 3 days of ^13^C labeling, compared with the CK, the FA application (FA1–FA4) significantly elevated ^13^C accumulation in all seedling organs, and the best promotion effect was observed under the FA2 treatment (Figure 5A). We further measured the organ ^13^C distribution rate under the different treatments. The ^13^C labeling results in Figure 5B show that the FA treatment (FA1–FA4) obviously decreased the leaf ^13^C distribution rate, and the FA2 treatment resulted in the lowest value, which was decreased by 17.14% compared with the CK. Moreover, compared with the CK, the root ^13^C distribution rate was also significantly influenced by the FA application. Diametrically opposite to the leaf ^13^C distribution rate, the root ^13^C distribution rate first increased and then decreased with the increasing FA concentration, and the highest value of the ^13^C distribution rate in the roots appeared in the FA2 treatment, which showed an increase of 52.78% compared with the CK.

We subsequently measured the gene expression of *MdSOTs* and *MdSUTs* in the roots. As presented in Figure 5C–K, with the increasing FA levels, the gene expression of *MdSOT1* and *MdSOT2* first increased and then decreased, and both reached the highest level under the FA2 treatment. Compared with the CK, the FA2 and FA3 treatments both significantly upregulated the *MdSOT3* gene expression; however, there was no significant difference in the *MdSOT3* gene expression between the FA2 and FA3 treatments. For the *MdSUT* gene expression, we discovered that the gene expression of *MdSUT1.1* and *MdSUT1.2* was also influenced (upregulated) by the FA treatment compared with the CK. In contrast, the gene expression of *MdSOT4*, *MdSUT2.1*, *MdSUT2.2*, and *MdSUT4.1* did not change significantly regardless of whether the FA treatment was conducted.

### 2.3. N Absorption and Metabolism

#### 2.3.1. NO_3_^−^ Ion Flow Rate in Roots

**Figure 6:**
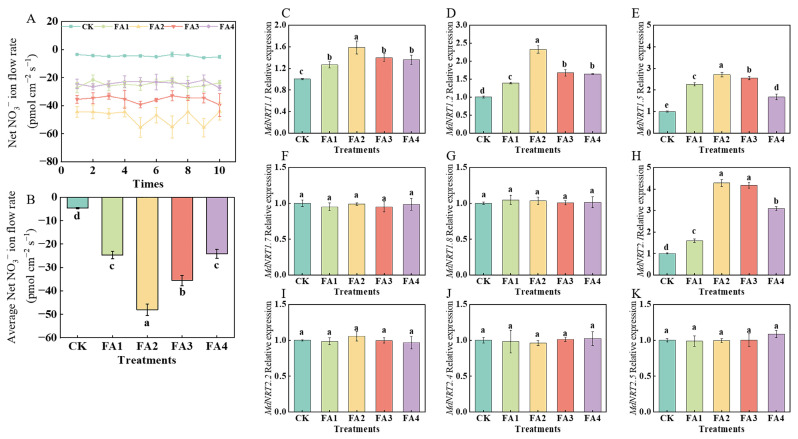
Net NO_3_^−^ fluxes for 10 min (**A**), the mean rate of NO_3_^−^ fluxes during the entire 10 min period (**B**), and *MdNRT* gene expression (**C**–**K**) in roots treated with CK (0 mg·L^−1^ fulvic acid), FA1 (60 mg·L^−1^ fulvic acid), FA2 (120 mg·L^−1^ fulvic acid), FA3 (180 mg·L^−1^ fulvic acid), or FA4 (240 mg·L^−1^ fulvic acid). Vertical bars on the histograms indicate standard deviations of three replications. Different letters indicate statistically significant differences (*p* < 0.05).

As shown in Figure 6A, regardless of the treatments, NO_3_^−^ tended to be absorbed due to the negative values of the NO_3_^−^ ion flow rate, and the average ion flow rate was significantly promoted by the FA treatment to varying degrees. The maximum average ion flow rate was observed under the FA2 treatment (Figure 6B).

#### 2.3.2. NRT Gene Expression in Roots

As shown in Figure 6C–K, the transcript levels of *MdNRT1.1*, *MdNRT1.2*, *MdNRT1.5*, and *MdNRT2.1* were significantly upregulated by exogenous FA treatment compared to the CK. The highest transcript levels of *MdNRT1.1*, *MdNRT1.2*, and *MdNRT1.5* were observed under the FA2 treatment. Compared with the CK, the FA2 and FA3 treatments both significantly upregulated the *MdNRT2.1* gene expression; however, there was no significant difference in the *MdNRT2.1* gene expression between the FA2 and FA3 treatments. Additionally, regardless of the treatment, the gene expression of *MdNRT1.7*, *MdNRT1.8*, *MdNRT2.2*, *MdNRT2.4*, and *MdNRT2.5* did not change significantly.

#### 2.3.3. N Metabolism-Related Enzyme

**Figure 7:**
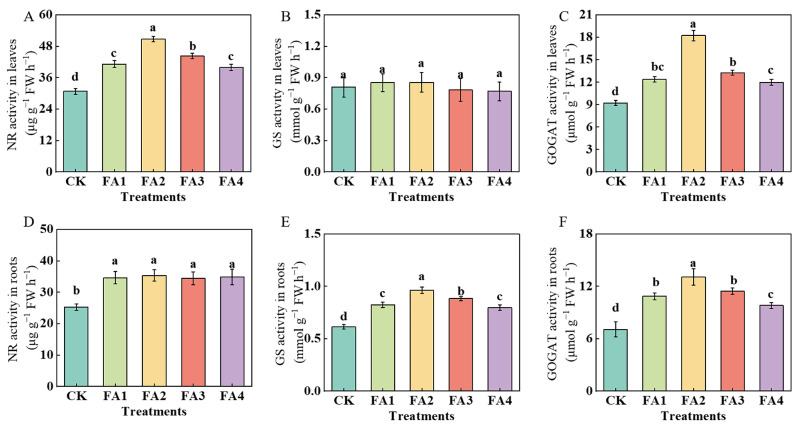
The activities of NR, GS, and GOGAT in leaves (**A**–**C**) and roots (**D**–**F**) treated with CK (0 mg·L^−1^ fulvic acid), FA1 (60 mg·L^−1^ fulvic acid), FA2 (120 mg·L^−1^ fulvic acid), FA3 (180 mg·L^−1^ fulvic acid), or FA4 (240 mg·L^−1^ fulvic acid). Vertical bars on the histograms indicate standard deviations of three replications. Different letters indicate statistically significant differences (*p* < 0.05).

As shown in Figure 7A, the leaf NR activity in the FA2 treatment was the highest, i.e., 65.21% higher than that in the CK. Additionally, although exogenous FA treatment (FA1–FA4) significantly elevated the root NR (nitrate reductase) activity compared to the CK, there was no significant difference in the root NR activity between them (Figure 7D). The GS (glutamine synthetase) activity was also measured. As shown in Figure 7B, the FA application had no significant effect on the leaf GS activity. Compared with the CK, the FA treatment elevated the GS activity in the roots, and the seedlings in the FA2 treatment group had the highest root GS activity, i.e., 56.93% higher than that in the CK. Moreover, the FA supply treatments also increased the GOGAT (glutamate synthase) activity in the leaves and roots (Figure 7C,F), and both activities were maximized under the FA2 treatment.

#### 2.3.4. ^15^N Accumulation and ^15^N Distribution Ratio

**Figure 8:**
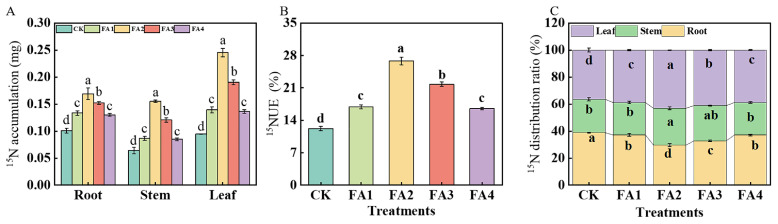
^15^N accumulation (**A**) and ^15^NUE of seedlings (**B**) as well as organ ^15^N distribution rate (**C**) treated with CK (0 mg·L^−1^ fulvic acid), FA1 (60 mg·L^−1^ fulvic acid), FA2 (120 mg·L^−1^ fulvic acid), FA3 (180 mg·L^−1^ fulvic acid), or FA4 (240 mg·L^−1^ fulvic acid). Vertical bars on the histograms indicate standard deviations of three replications. Different letters indicate statistically significant differences (*p* < 0.05).

The ^15^N labeling results shown in Figure 8A show that regardless of plant organs, the FA2 treatment resulted in the highest ^15^N accumulation, which was elevated by 1.70 (root), 2.67 (stem), and 2.78 (leaf) times those of the CK, respectively. ^15^NUE was measured according to the method reported by Xu et al. [17]. Compared with the CK, all the FA treatments elevated the seedling ^15^NUE, and the highest ^15^NUE was found under the FA2, followed by the FA3 (Figure 8B). Analysis of the organ ^15^N distribution ratio was also performed. As shown in Figure 8C, the FA application (FA1–FA4) significantly decreased the root ^15^N distribution ratio but increased the leaf ^15^N distribution ratio. The lowest root ^15^N distribution ratio and highest leaf ^15^N distribution ratio were both observed under the FA2 treatment.

## 3. Discussion

This study was conducted to analyze the effects of fulvic acid (FA) on the growth and nitrogen utilization efficiency (NUE) in M9T337 seedlings. Although it has been widely documented that FA application could promote plant growth and its nutrient absorption ability [9,11], its effects on the growth and NUE in apples remain unclear, particularly from the perspective of C and N metabolism regulation.

### 3.1. FA Promoted Photosynthesis and the Transport of Photosynthates from Leaves to Roots

FA plays an important role in agricultural production in China due to its vital function in the promotion of plant growth under normal or stress conditions [9,19]. The findings observed in this experiment showed that the FA application elevated the dry weight of seedling organs compared with the CK, and the highest seedling dry weight occurred under the FA2 treatment (Figure 1A). As the main plant organ to absorb nutrients, the response of roots to exogenous substance application may be the initial driving force for promoting plant growth [20]. To better explain why FA application has a positive effect on seedling growth, we further analyzed seedling root morphology parameters. The results indicated that the FA application had a favorable promotion effect on the total root length and root surface area compared with the CK (Figure 1B,C). A possible reason for this finding is that FA application behaves as an exogenous indole acetic acid (IAA), which may regulate root growth and morphology via cell division and expansion [13]. Moreover, the higher ^15^NUE under the FA treatments may also be beneficial to the root growth, because N is a macroelement necessary for plant growth [21]. Yu et al. [22] reported that the effect of FA on wheat root growth under low N stress is affected by its concentration, showing a dramatic dose dependence. The results of this study showed that the seedling biomass also showed an obvious dramatic dose dependence and peaked under the FA2 treatment, possibly because FA application at an appropriate concentration to the root system may induce a eustress state (positive stress), thus promoting seedling growth [23]. Compared with the FA2, the higher dose (FA3 and FA4) both decreased the root growth of seedlings, the reason might be that the higher dose breaks the balance of the eustress state, and increased the level of reactive oxygen species (ROS), which could inhibit the growth of the root. Moreover, the change in ^15^NUE between the FA2 and the higher dose (FA3 and FA4) may also be beneficial in explaining the difference in the root growth, because N is a macroelement necessary for plant growth [1].

The photoassimilates used for root growth and development are directly dependent on the leaf-to-root translocation of photoassimilates [4]. Therefore, the change in photosynthesis, photosynthesis media C (carbon) assimilation as well as the leaf-to-root translocation of photoassimilates could also be beneficial in explaining the effects of different FA levels on seedlings’ growth. The results of this study indicated that the content of chlorophyll was obviously elevated under the FA application and peaked under the FA2 treatment (Figure 2A), which was in line with the results obtained by Wang et al. [19]. Moreover, previous studies indicated that enhancing the N absorption capability of plants is conducive to photosynthesis-mediated C fixation in plants, which might be due to the close relationship between N and the chlorophyll content [24]. Therefore, the highest N absorption and N distribution rate in the leaves under the FA2 treatment may be another explanation for the increase in the chlorophyll content in the leaves (Figure 8A,C). In this experiment, the changes in the *P*_n_ and *G*_s_ in the leaves among the CK and the different exogenous FA applications were also analyzed. Compared with the CK, the values of the leaf *P*_n_ and *G*_s_ were both significantly elevated under the FA application (Figure 2B,C). As described by Lawlor and Cornic [25], the synthesis of photosynthetic assimilates is closely related to CO_2_ absorption by leaves. A higher *G*_s_ under the FA treatment indicates that the leaf CO_2_ absorption ability was elevated, which may be beneficial in explaining the difference in the total seedling ^13^C accumulation among various treatments. As an important indicator to evaluate the photosynthetic efficiency of seedlings, the result revealed that the FA treatments significantly increased the *ETR* in the leaves, which was consistent with the findings reported by Fan et al. [26]. These results indicated that FA treatment could strengthen the photosynthetic efficiency of seedlings. In line with the results obtained by Wang et al. [27] and Liu et al. [28], we also observed that the *P*_n_, *G*_s_, and *ETR* of the leaves showed an obvious dramatic dose dependence, indicating that an appropriate FA supply (FA2) could maximize the photosynthetic efficiency and electron transfer in leaves. These benefits also provide a basis for the enhancement of photosynthesis media C (carbon) assimilation.

In higher plants, the synthesis, transport, and distribution of photosynthetic products are important physiological processes [29,30]. The CO_2_ absorbed by leaves can be assimilated through the actions of C metabolism-related enzymes [7,9]. We observed that the activities of Rubisco treated with FA were obviously greater than those treated with the CK (Figure 2F), which meant that the FA treatment enhanced the capacity of photosynthetic assimilate synthesis to varying degrees. The synthesis of sorbitol and sucrose in the leaves and their transport from leaves to other organs has a very positive effect on the various physiological and biochemical processes in plants (such as organ construction) [31]. In this study, compared with the CK, the FA application increased the S6PDH, SPS, and SS activities in the leaves (Figure 3A–C), suggesting that the sorbitol and sucrose synthesis in the leaves were enhanced when the seedlings were exposed to the FA treatment conditions. However, we discovered that the FA application obviously decreased the sorbitol and sucrose contents in the leaves, while a significant elevation was observed in the roots (Figure 4), indicating that the leaf-to-root translocation of photoassimilates was enhanced under the FA treatment, and these results might also contribute to explaining the difference in the root ^13^C accumulation between the CK and the FA treatments presented in our ^13^C labeling experiment to a certain degree (Figure 5A). In contrast, the concentration of sorbitol and sucrose in the leaves treated with the FA3 and FA4 treatments was higher than that of the FA2, indicating that the leaf-to-root translocation of photoassimilates was reduced. Usually, a negative correlation between the amount of soluble sugars in leaves and the rate of photosynthesis is expected. Therefore, the accumulation of sorbitol and sucrose in the leaves might be conducive to explaining why the seedlings treated with the FA3 and FA4 treatments had a poorer photosynthesis performance than that of the FA2 treatment. *MdSOTs* and *MdSUTs* play a vital role in sorbitol and sucrose transport [32]. We observed that the FA treatment obviously upregulated the root *MdSOT1*, *MdSOT2*, *MdSOT3*, *MdSUT1.1*, and *MdSUT1.2* gene expression (Figure 5C–K), thus enhancing the leaf-to-root translocation of photoassimilates. Moreover, the activities of SDH, SuSy as well as FRK and HK in the root were also elevated under the FA treatments (Figure 3D–G), indicating that the process of sugar metabolism in the root was promoted under the FA treatments. The elevation in the sorbitol and sucrose contents as well as the enhanced sugar metabolism in the FA-treated seedling roots may also explain why the seedlings displayed a better root growth and a higher ^15^NUE in the FA-treated condition. Bayat et al. [24] observed that the application of FA could promote the absorption of potassium in plants, and the elevation in the potassium content could be beneficial to the synthesis and transport of photosynthetic products as well as the elevation in sugar metabolism enzyme activities [33]. Therefore, we conjectured that the enhanced sugar transport was closely related to the promotion of potassium absorption. This study demonstrated that the role of FA in promoting seedling root growth can be expressed through the optimization of the assimilation, distribution, and utilization of photosynthetic products. Moreover, the efficient leaf-to-root translocation of carbohydrates encouraged root development, which may provide the basis for efficient N uptake and utilization by apple seedlings.

### 3.2. FA Optimized N Absorption, Assimilation, and Distribution in M9T337 Seedlings

N is a macroelement necessary for plant growth [1,24,34], and the role of FA application in promoting N absorption and assimilation in plants has been widely documented [9,24]. The results of our ^15^N labeling showed that the FA application elevated the seedling ^15^NUE (Figure 8B) to varying degrees, indicating that the seedling ^15^N absorption was enhanced under the FA-treated conditions. Except for the optimization of the root morphology and higher root activity under the various levels of FA application (Figure 2B–D), the enhancement in the N intake (the first crucial step that may lead to differences in plant N absorption as well as N assimilation) may also explain why the FA-treated seedlings had higher NUE values. The results obtained by Xing et al. [35] in maize, Anwar et al. [36] in cucumber as well as Tavares et al. [37] in rice plants showed that the efflux or influx of N (NO_3_^−^ or NH_4_^+^) may be directly reflected according to the positive or negative values of the NO_3_^−^ and NH_4_^+^ ion flux rates. The NMT results in this study also presented a NO_3_^−^ influx into the roots regardless of the treatments employed in this study, according to the NO_3_^−^ flow rate values, and the NO_3_^−^ influx rate was elevated by the FA treatment (Figure 6A,B), indicating that FA can also significantly enhance the N influx, thus promoting N absorption in seedlings. The results obtained by Peng et al. [38] in soybeans showed that the decreased sucrose content in roots may promote root nutrient absorption ability. Moreover, Xu et al. [33] noted that the promotion of sugar metabolism-related enzyme activities in roots may be an important explanation for the change in plant root growth because the process of sugar metabolism could provide the energy and intermediates for root growth. Therefore, the promotion of the sorbitol and sucrose transport from the leaves to roots as well as the elevation in the SDH, SuSy, FRK, and HK activities in the roots under the FA2 treatment in this study might be an important reason to explain why the seedlings treated with the FA2 had a higher ^15^NUE than other treatments. Apart from the indices mentioned above, the changed NRT gene expression levels are also closely related to the elevation in plant N absorption to a certain degree [5]. Similar to the results obtained by Jannin et al. [39] in *Brassica napus*, the FA treatment obviously upregulated the *MdNRT1.1*, *MdNRT1.2*, *MdNRT1.5*, and *MdNRT2.1* expression in the roots (Figure 6C–K). The NO_3_^−^ absorbed by the roots can remain in the roots or be transported to other organs, where it is then assimilated through the actions of a series of enzymes [40,41], and the results obtained by Gao et al. [9] in maize showed that FA application could promote plant N metabolism by regulating the activities of NR and GS. In line with the results obtained by Yu et al. [22] and Vaccaro et al. [42], the results of this study showed that the FA application significantly promoted the activities of NR, GS, and GOGAT in the leaves or roots to varying degrees (Figure 7), indicating that the N assimilation capacity of the seedlings was enhanced under the FA application, especially under the FA2 treatment. The promotion of photosynthesis caused by the FA2 application may be an important explanation for why FA treatment can strengthen N assimilation in seedlings because photosynthesis can provide the basis of material and energy, such as C skeletons and ATP, which are required for N assimilation [4,43]. In contrast, a poorer photosynthesis performance and lower photoassimilates in roots could be one of the important reasons to explain why the seedlings treated with FA2 had a higher ^15^N absorption and assimilation efficiency than that of the FA3 and FA4 treatments.

The mechanism of the simultaneous promotion of C and N metabolism in the enhancement of plant nutrient absorption has been widely acknowledged [44]. Xu et al. [17] reported that the elevation in nitrogen distribution in leaves is beneficial to improving the efficiency of photosynthetic nitrogen utilization and photosynthesis media C assimilation. Han et al. [45] also indicated that increasing the leaf N distribution rate could improve the photosynthetic efficiency, which in turn promotes nutrient absorption by roots. The ^15^N labeling results showed that the lowest leaf ^15^N distribution ratio was observed under the CK, while the leaf ^15^N distribution ratio of the seedlings in the FA treatments was obviously elevated (Figure 8C), suggesting that the root-to-leaf translocation of N was improved under the FA application. Xu et al. [4] and Chen et al. [46] both observed that the change in the *NRT1.5* expression is closely associated with the change in the root-to-leaf translocation of NO_3_^−^. In this study, the root-to-leaf translocation of the N and *NRT1.5* expression were both optimized under the FA treatments, and the best promotion effects were both observed under the FA2 treatment. Therefore, the elevation in the leaf ^15^N distribution ratio under the FA treatments may be related to the upregulation of the *MdNRT1.5* expression induced by FA (Figure 6E).

## 4. Materials and Methods

### 4.1. Plant Material and Growth Conditions

M9T337 seedlings were used in the experiments and were grown in a chamber under natural light and 22–26 °C day and 5–10 °C night conditions, with a relative humidity (RH) of 55–65%. When the seedlings grew to approximately 12 cm, M9T337 seedlings with similar appearances were selected and transplanted into plastic basins (with a size of 40 cm × 30 cm × 15 cm). Eight seedlings were grown in each basin. Then, six liters of 100% strength Hoagland’s nutrient solution was added to each basin. Notably, to improve adaptation to the nutrient solution, an equal volume of half-strength nutrient solution was added for 7 days before the application of the full-concentration Hoagland’s nutrient solution. The nutrient solution was changed as often as once every three days. Hoagland’s solution was prepared according to Hoagland and Arnon [47].

### 4.2. Experimental Design and Sampling

The formal experiment was conducted 15 days after the application of the full-concentration Hoagland’s nutrient solution using hydroponics. FA purchased from Bio Aladdin (Shanghai, China) was added to the nutrient solution during every replacement of the nutrient solution. There were five treatments, namely, CK, FA1, FA2, FA3, and FA4, which represented the five FA levels (0, 60, 120, 180, and 240 mg·L^−1^). Each treatment contained thirty-six plastic basins, eight seedlings in each basin, twelve basins pooled as a repeat, and each treatment was replicated three times. To exclude the influence of different FA levels on the ^15^N and ^13^C natural abundances in seedling organs, each repeat was divided into two groups: one group was used for ^15^N and ^13^C labeling as well as the measurement of ^15^N and ^13^C abundances (labeling group), and the other group (normal group) was used for measuring other indices. The experimental treatment lasted for 30 days. After treatment 30 days later, seedlings were harvested. The concentrations of nutrient elements in this experiment were as follows: 6 mM K_2_SO_4_, 5 mM Ca(NO_3_)_2_, 2 mM MgSO_4_, 1 mM NaH_2_PO_4_, 0.1 mM EDTA-Fe, 37 µM H_3_BO_4_, 9 µM MnCl_2_·4H_2_O, 0.76 µM ZnSO_4_·7H_2_O, and 0.3 µM CuSO_4_·5H_2_O.

### 4.3. Analysis of Dry Matter Weight

The seedlings were harvested and divided from top to bottom into leaves, stems, and roots. Following the details reported by Xu et al. [17], the samples were rinsed and dried. Subsequently, the dry weight of all plant organs was measured using a 1/1000 electronic balance.

### 4.4. Analysis of Root Morphology Parameters and Root Activity

After rinsing with deionized water, seedlings (randomly selected from the normal group of each treatment) were prepared to measure the total root length and total surface area using WinRHIZO v.2012b (Regent Instruments Canada Inc., Montreal, QC, Canada). The value of root activity under different treatments was also measured following the details reported by Chen et al. [48].

### 4.5. Photosynthetic Parameters

In this study, the *P*_n_ (net photosynthetic rate) and *G*_s_ (stomatal conductance) were measured using the LI-6400XT portable photosynthesis system (LI-COR, Lincoln, NE, USA), and the details of the measurements followed the steps reported by Xu et al. [17]. Moreover, the leaves (used for the measurement of the *P*_n_ and *G*_s_) were selected to measure the chlorophyll fluorescence parameters using a pulse-modulated chlorophyll fluorescence meter (PAM 2500, Walz, Germany) during the same period, and every measurement was replicated three times. The method described by Porra [49] was used to measure the leaf chlorophyll content.

### 4.6. Measurement of Enzyme Activities

The measurement of the Rubisco (Ribulose-1,5-biphosphate carboxylase-oxygenase) activity was conducted according to Hu et al. [50]. The methods reported by Huber and Israel [51] were used for the determination of the sucrose synthase (SS) and sucrose phosphate synthase activities (SPS). Subsequently, the methods described by Berüter [52] were adopted in this study for the determination of the sorbitol 6-phosphate dehydrogenase (S6PDH) activity. The method reported by Rufly and Huber [53] was adopted in this experiment to analyze the activity of sorbitol dehydrogenase (SDH). The activities of fructokinase (FRK) and hexokinase (HK) were measured according to the method of Li et al. [54]. The nitrate reductase (NR), glutamine synthetase (GS), and glutamate synthase (GOGAT) activities were estimated based on the methods outlined by Hu et al. [55].

### 4.7. Measurement of Sorbitol and Sucrose Concentrations

The concentrations of sorbitol and sucrose in the leaves and roots were measured in this experiment. Following the details reported by Tian et al. [7], the sample was prepared for the determination of the sorbitol and sucrose concentrations using the filtrate with liquid chromatography methods [56].

### 4.8. ^13^C and ^15^N Labeling Method and Isotope Analysis

The basins of the labeling group in each treatment were selected for ^15^N labeling. Ca(^15^NO_3_)_2_ (0.01 g per basin; abundance, 10.14%) was added to the nutrient solution at every change of solution. Each basin contained 0.1 g Ca(^15^NO_3_)_2_ in total during the whole experimental treatment period.

The ^13^C labeling experiment was activated at 3 days before the end of the treatment period. In brief, labeling chambers made of Mylar plastic bags and brackets were prepared to cover and seal the seedlings of the label group of each basin. Each basin corresponded to one labeling chamber. A small beaker with 2 g of Ba^13^CO_3_ together with fans and reduced iron powder was placed into the chamber. After turning on the fan and sealing the chamber, ^13^C pulse labeling was performed. Considering the maintenance of the ^13^CO_2_ concentration, we injected hydrochloric acid into the beaker every 30 min. The concentration of hydrochloric acid used in the ^13^C pulse labeling was set to 1 mol L^−1^. The process of ^13^C pulse labeling lasted for 4 h.

The seedlings of the labeling group of each treatment were divided into three parts, from bottom to top: roots, stems, and leaves. The preparation before the determination of the ^15^N and ^13^C abundance and the calculation of ^13^C and ^15^N abundance both followed the method reported by Xu et al. [17]. Subsequently, the samples prepared for the measurement of ^15^N abundance were transferred to the laboratory, and then, the measurement was performed by a MAT-251-Stable Isotope Ratio Mass Spectrometer. The samples used for ^13^C abundance determination were measured by a DELTAVplusXP advantage isotope ratio mass spectrometer. The computational formulas of ^15^N- and ^13^C-related indices are shown as follows:

Calculation of ^15^N:

Ndff (%) = [(abundance of ^15^N in plant − natural abundance of ^15^N)/(abundance of ^15^N in fertilizer − natural abundance of ^15^N)] × 100% (1)

^15^N absorbed by each organ = Ndff (%) × total N content (mg) (2)

Calculation of ^13^C:

Abundance of ^13^C: F*_i_* (%) = [(δ^13^C + 1000) × R_PBD_]/[(δ^13^C + 1000) × R_PBD_ + 1000] × 100% (3)

R_PBD_ is a constant value that represents the standard ratio of carbon isotopes. The value of R_PBD_ is set to 0.0112372.

C content of each organ:

C_i_ = organ dry matter (g) × organ total carbon content (%) (4)

Content of ^13^C in each organ:

^13^C_*i*_ (mg) = [C_i_ × (F*_i_* − F*_nl_*)/100] × 1000 (5)

The value of F*_nl_* represents the ^13^C natural abundance of each organ:

^13^C partitioning rate: ^13^C (%) = (^13^C*_i_*/^13^C _net absorption_) × 100% (6)

### 4.9. Determination of Root NO_3_^−^ Flow Rate

To better evaluate the N absorption ability of roots, we used a noninvasive microtest system (NMT Physiolyzer, Younger USA Sci. &Tech. Corp., Amherst, MA, USA) to explore the effects of FA treatment on the root NO_3_^−^ flow rate. The preparation and steps of the measurement followed the methods presented by Xu et al. [17]. The measurement process lasted for 10 min, and the data were examined with MageFlux (imFluxes v 2.0). The efflux or influx of NO_3_^−^ was analyzed according to the positive and negative values of the NO_3_^−^ flow rate.

### 4.10. RNA Isolation and qRT-PCR Analysis

Following the RNAiso Plus manufacturer’s guidelines (Takara, Otsu, Shiga, Japan), total RNA was extracted from the samples. Subsequently, the concentration and purity of the total RNA was determined. Then, ReverTra Ace^®^ qPCR RT Master Mix with gDNA Remover (TOYOBO, Osaka, Japan) was used to synthesize cDNA. Relative gene expression was analyzed via RT-qPCR on a LightCycler 96 (Roche, Basel, Switzerland) using TranStart Top Green qPCR SuperMix (TransGen Biotech, Beijing, China). The *MdActin* gene was used as the internal control. Relative expression was calculated using the 2^−△△CT^ method [57]. The details of the primers are listed in Supplementary Table S1. Each treatment contained three technical and three biological replicates.

### 4.11. Statistical Analysis

Microsoft Excel was used for the collection of data measured in this experiment, and SPSS 21.0 (SPSS, Inc., Chicago, IL, USA) was used to analyze the data. The analysis of data was performed using a one-way analysis of variance (ANOVA) and a post hoc test (Duncan’s). Significant differences were considered at a probability level of *p* < 0.05.

## 5. Conclusions

**Figure 9:**
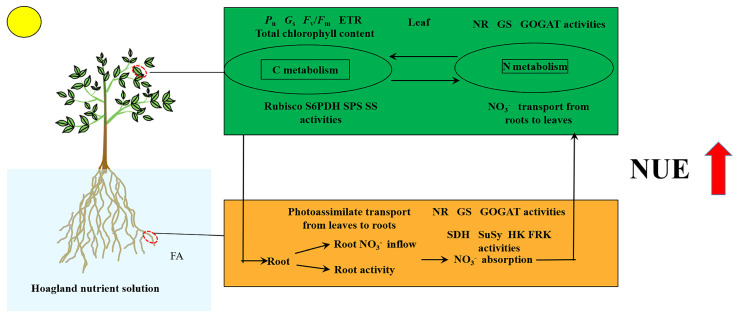
Schematic model displaying the role of FA on the promotion of NUE in apple seedlings.

In conclusion, the seedlings exposed to the FA treatment displayed the following characteristics (Figure 9): (i) significantly promoted root growth and higher root activity; (ii) the promotion of leaf-to-root translocation of photoassimilates; (iii) strengthened root NO_3_^−^ ion flow rate and *MdNRT* gene expression; (iv) elevated N metabolism-related enzyme activity; (v) more rational distribution of N in seedlings; and (vi) substantial increase in the NUE. Overall, this study offers fresh perspectives into the promotion of apple plant growth and NUE caused by FA application via the regulation of C and N metabolism.

## Data Availability

Data are contained within the article and Supplementary Materials.

## Supplementary Materials

The following supporting information can be downloaded at: https://www.mdpi.com/article/10.3390/plants12233937/s1, Table S1. qRT-PCR primers.
