# Supplementary material for: Effects of Fulvic Acid on Growth and Nitrogen Utilization Efficiency in M9T337 Seedlings
Source: Plants (Basel). 2023 Nov 22;12(23):3937. doi: 10.3390/plants12233937 (PMC10708164; doi:10.3390/plants12233937)
Supplement: Supplementary file 1 [file plants-12-03937-s001.zip › plants-2693321-supplementary.pdf]

Table S1. Primer sequences for PCR.

Gene	Forward Primer Sequence (5′→3′)	Reverse Primer Sequence (5′→3′)
MdACTIN	TGGTGTCATGGTTGGTATGG	CCGTGCTCAATGGGATACTT
MdNRT1.1	TTGGCTCTGACCAGTTCGAC	ACGGTACTGTCTTGTGCCTG
MdNRT1.2	TGTTGACTGGAGAAGCCGAC	GTGACATCGTTTGCCGCTTT
MdNRT1.5	GTGGTCATGAAGTTCTCGACAGAG	TCAGACTTTCAAGTTTCCTTGATCAT
MdNRT1.7	AGTTTCACGGTGACTGTGCTG	ACGTTGCGCACAAAACAGAA
MdNRT1.8	TGGCAATATTGGGCAAGCTG	CGTAATGGTAACACCAGTATGTGC
MdNRT2.1	TTGAGAGTTTGACAAGATAAACGCA	AACTGACGGGTGGGGAAATC
MdNRT2.2	TCGCTCTGGGGATCCATACT	TGCCTCGTGGAGTGTCATTT
MdNRT2.4	GCTGGGGGTTACATAGCAGTTAGG	CCATTAACCAGTCCAATAATCTTACCA
MdNRT2.5	TGTTGGTCCCACTTCTACCG	TGTCACCAGCAGTTCCCATC
MdSUT1.1	GTGGTAATTTACCGGCATTTGTCG	AAGCTAGAGGCCGTAGGGCAAG
MdSUT1.2	CGAAGACAGAAAGTGTGTGTCCTG	GTAGGGGGTGAGGAGGGAGAG
MdSUT2.1	CAGATGGTTTCCTTTCTTGTTGAG	TGCTGATACATAGTGAGTGGAACCT
MdSUT2.2	TTCCTTTTGAGTAGAGCTTGCTGTG	CTCAAGACCATGTTGTTGGGGAT
MdSUT4.1	TGATTTACCTCCAGTTGGCATTG	GCCAAGTCCCAAAGACTCAATTC
MdSOT1	GACAGAACCTCAGACTCCAAAG	TGACCAGAGACCTGAACGATA
MdSOT2	CGTATCCAACTATGCCTTCTCC	GACACCGACGGCAAGAATAA
MdSOT3	GCCGGTACTCTAAACATCTACTC	GAAGATGACTCCCGCAAGAA
MdSOT4	CTCGTGGTGATGGTGGCACCAGTG	CAGAGATGCATAATACGACTCACTATAGG<br>GAGATCTAATACGAAATGTTTTAT
